# Dyadic coping and well-being in early-stage dementia couples

**DOI:** 10.3389/fpsyt.2025.1613215

**Published:** 2025-09-19

**Authors:** Peter Muijres, Guy Bodenmann, Fridtjof W. Nussbeck, Josef Jenewein

**Affiliations:** ^1^ Department of Psychology, Clinical Psychology for Children/Adolescents and Couples/Families, University of Zurich, Zürich, Switzerland; ^2^ Department of Psychology, Methods for Intensive Data in Psychology, University of Konstanz, Konstanz, Germany; ^3^ Private Clinic Hohenegg AG, Meilen, Switzerland

**Keywords:** dyadic coping, anxiety, depression, quality of life, dementia, couples

## Abstract

**Introduction:**

This study examined the relationship between dyadic coping (DC) and anxiety, depression, and quality of life in 37 couples facing early-stage dementia (ESD).

**Methods:**

The Actor-Partner Interdependence Model was used for the analyses.

**Results:**

The study revealed significant actor effects of DC on these outcomes, particularly for quality of life domains. Subjects with ESD providing more delegated DC and engaging more in common DC showed less anxiety. Caregiving partners experienced lower distress when engaging in emotion-focused common DC. Interestingly, positive DC from caregiving partners was associated with lower quality of life in patients.

**Discussion:**

We found more actor effects than partner effects, suggesting that DC behaviors primarily impact one’s own well-being, rather than the partner’s well-being. The different patterns of DC effects on subjects with ESD and their partners reflect a need for tailored interventions that consider the unique needs of each partner in couples facing ESD.

## Introduction

Worldwide, more than 50 million people are estimated to be living with dementia, making it one of the most pressing public health concerns today (1). In Switzerland alone, approximately 110,000 individuals are currently affected by dementia, a figure projected to rise to 190,000 by 2030 (2). According to the Federal Office of Public Health (2), dementia prevalence increases significantly with age, affecting about 12% of individuals aged 80 to 84 years.

Early-stage dementia (ESD) entails memory loss, orientation, difficulties with problem-solving and judgment, with community involvement, household and leisure activities, and personal care (3). A reduced ability to perform daily tasks (4) and to participate in social activities and conversations (5) is associated with anxiety and depression. A reduced quality of life, anxiety and depression in people with ESD may also be associated with the distress caused by an awareness of their cognitive decline (6), as well as a struggle with the loss of autonomy and a fear of the future (7). The heightened psychological distress that accompanies ESD impacts both affected subjects and their partners (6, 7).

Due to the progressive cognitive decline, which impairs the ability to manage daily tasks and maintain independence, persons with ESD depend increasingly on support. Within couples, romantic partners often assume the role of primary caregiver, providing both physical and emotional assistance (7). Several studies have shown that caregiving stress and uncertainty exacerbate emotional strain on caregiving partners (22). They often experience anxiety and depression as a result of caregiving burdens, which compromise their mental health and well-being (8). The quality of the provided care is closely linked to the well-being of both the caregiving partners and the affected subjects. When caregiving partners experience high levels of stress or poor mental health, their ability to offer effective support diminishes, leading to a deterioration of mental health for both parties. Dyadic Coping (DC), or how couples cope with stress together, plays a pivotal role in relationship quality and well-being of both partners in the context of dementia (7, 9, 10).

DC is conceptualized in the most differentiated way in the Systemic Transactional Model (STM) of stress and coping within couples (11, 12). DC characterizes the interactive process in which one partner signals stress through verbal, paraverbal, or nonverbal cues, while the other partner responds to these signals through verbal and/or nonverbal reactions (11, 13, 14). DC helps restore homeostasis in the relationship by fostering mutual understanding and support during challenging times and is a significant predictor of relationship functioning (15).

DC comprises several dimensions. Stress communication represents the extent to which a stressed person communicates their stress to their partner and seeks support (e.g. ‘I ask my partner to do things for me, when I have too much to do’). Supportive DC describes one partner’s efforts to assist the other in their coping efforts (e.g. showing empathy and understanding towards one’s partner). Delegated DC involves efforts of one partner to relief the stress of the other partner by taking over tasks and responsibilities (e.g. ‘When my partner feels he/she has too much to do, I help him/her out). Negative DC includes hostile, ambivalent or superficial actions or words (e.g. ‘I do not take my partner’s stress seriously’). Common DC describes both partners experiencing stress and their joint efforts to cope with it. Common DC can be emotion-focused or problem-focused (e.g. ‘We engage in a serious discussion about the problem and think through what has to be done’) (11, 16). DC is linked to levels of anxiety and depression in both persons with ESD and their caregiving partners, improving their overall quality of life by enhancing emotional resilience and relationship satisfaction (17, 18).

Despite growing recognition of the importance of DC in chronic illness (19), research specifically addressing its role in ESD remains limited (20, 21). Available studies highlight the positive impact of DC on both affected subjects and caregiving partners. DC was found to play a significant role on the well-being of both partners within couples facing dementia (10, 22). These findings align with the broader literature on chronic illness, which assert that positive DC, including supportive DC, delegated DC and common DC, improves well-being of couples facing various health conditions (e.g. 15, 18, 23–28). Given that most research is addressing chronic physical conditions (19), the lack of studies specifically focusing on DC in couples facing dementia beckons for more targeted studies to better understand the interdependence between DC and well-being in these couples.

**Figure 1:**
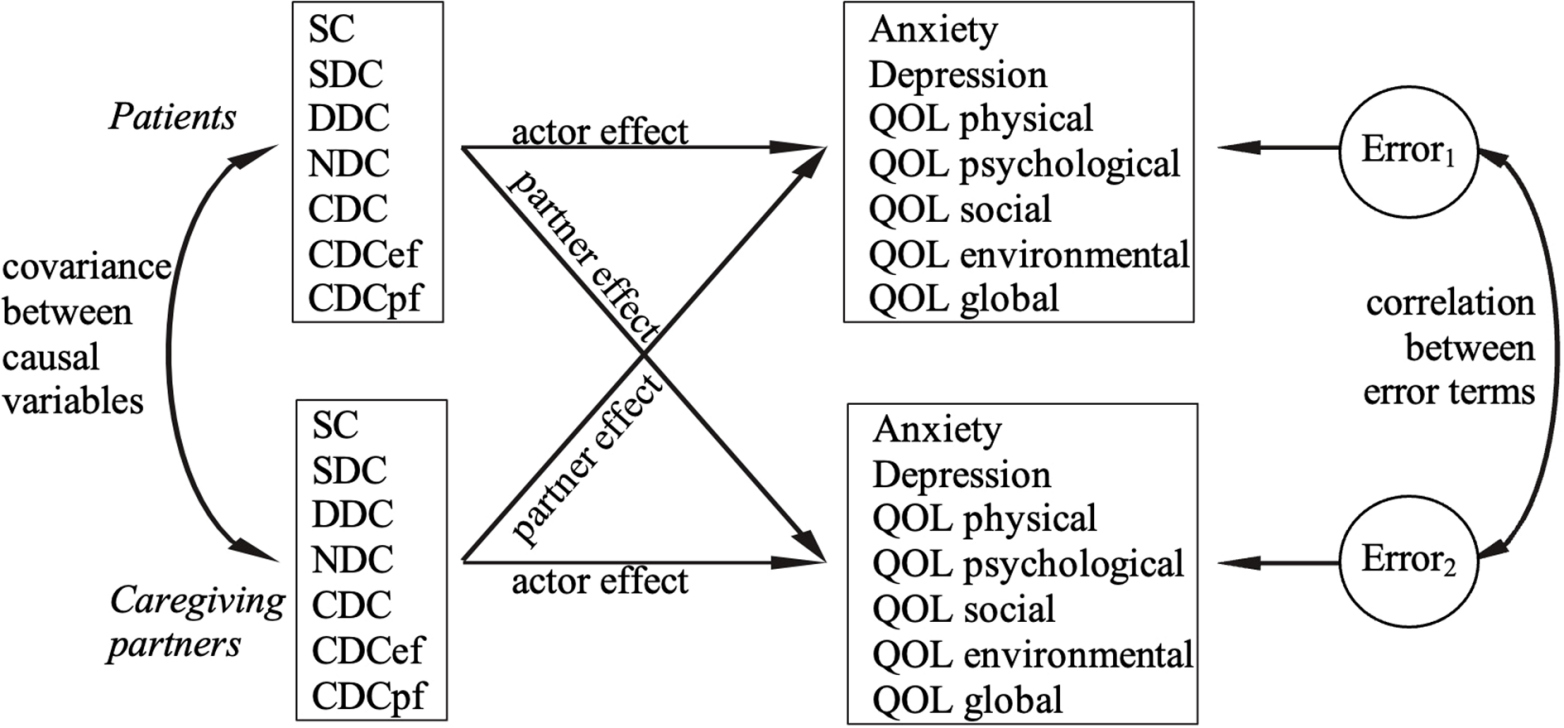
The relations between provided DC and one’s own anxiety, depression and quality of life and that of the other member of the dyad. DC, dyadic coping; SC, stress communication; SDC, supportive DC; DDC, delegated DC; NDC, negative DC; CDC, common DC; CDCef, emotion-focused CDC; CDCpf, problem-focused CDC; QOL, quality of life.

Considering the interdependence of dyads facing ESD, the current study examines, how domains of DC of each member of the dyad relates to anxiety, depression, and quality of life in both members. We expected that subjects with ESD and their partners who engage more in positive DC, i.e. more supportive DC, delegated DC, common DC (including emotion-focused and problem-focused common DC, and less in negative DC will report lower levels of anxiety and depression, and better quality of life on all domains. **Figure 1** details the research questions related to the effects of each individual’s DC efforts on outcome variables of both partners.

## Methods

### The current study

The current study was part of the DTD-research project ‘Dignity Therapy and Dementia’, in which the feasibility, acceptance and benefits of dignity therapy in people with dementia and their relatives was studied (29). In the current study, our goal was to understand how DC efforts by persons with ESD relate to anxiety, depression, and the domains of quality of life of themselves, as well as of their caregiving partners. Vice versa, we aimed to understand what effects the DC efforts of the caregiving partners have on outcomes in themselves, as well as in the persons with ESD.

### Participants

Persons with ESD were recruited at the University Geriatric Outpatient-Center Waid, Switzerland between March 2019 and October 2020. Inclusion criteria were (1) adult persons with (2) a diagnosis of very mild or mild dementia corresponding with a Clinical Dementia Rating of between 0.5 and 1.5 (30), who were (3) in a close and committed relationship with a partner unaffected by chronic health conditions. The score of zero on the Clinical Dementia Rating indicates no dementia; 0.5 a questionable dementia; 1.0 a mild dementia; 2.0 a moderate dementia; and 3.0 a severe dementia. Couples were excluded if (1) one partner had insufficient knowledge of the German language or (2) if the caregiving partner was affected by a chronic illness. All couples in the sample were heterosexual, although heterosexuality was no inclusion criterion.

Among the 37 heterosexual couples, 36 were married (97%) for an average duration of 46.9 years (range 5.0–67.0, *SD* = 13.9). Of the 37 subjects with ESD, 23 persons were male (62%) and 14 persons were female (38%). The average age of the patients was *M* = 78.8 years of age (range 63–89, *SD* = 5.9) and of the partners *M* = 76.8 years of age (range 59–90, *SD* = 7.2). **Table 1** shows additional sociodemographic data.

**Table 1:** Participants’ basic demographics (N = 37 couples).

Demographic variable		Patients with ESD (*n* = 37)		Partners (*n* = 37)	
		*n*	%	*n*	%
Gender	Female	14	37.8	23	62.2
	Male	23	62.2	14	37.8
Occupation	Part-time employed	2	5.4	8	21.6
	Full-time employed	0	0	7	18.9
	Pensioner	33	89.2	19	51.4
	Housekeeper	2	5.4	3	8.1
Relationship status	Married	36	97.3	36	97.3
	Unmarried	1	2.7	1	2.7
Education level	Compulsory education	4	10.8	3	8.1
	Apprenticeship	14	48.6	23	62.2
	Secondary school	5	13.5	0	0
	Technical college	8	21.6	5	13.5
	University	6	16.2	6	16.2

### Procedure

The consulting physicians at the study site made eligible subjects with ESD aware of the study. With their permission, contact details of these participants were passed on to the study coordinator, who was the first author. The study coordinator then first contacted them by telephone and informed them about the study and the use of questionnaires. Couples interested in participating, were sent a study information folder, and a personal information visit at home was planned.

The information visit provided ample opportunity to ensure that both members of the couple were comprehensively informed about all aspects of the study procedure. Both signed an informed consent form before completing the baseline assessment. During data collection, the study coordinator remained available for assistance and verified an accurate understanding and completion of items in case of doubt.

### Measures

#### Dyadic coping inventory

The Dyadic Coping Inventory (DCI, 3, 31) is a 37-item self-report instrument measuring DC behavior on a 5-point Likert-type scale ranging from 1 (*hardly ever*) to 5 (*very often*). Both, the subjects with ESD and their partners filled out the DCI. Each partner rated the levels of DC they provided to and received from the other partner.

To examine how the use of DC affects the well-being of both partners, seven DCI subscales were used in this study to tap into the dimensions of DC, that each partner provided. The subscales include: expressed stress communication (four items), supportive DC (five items), delegated DC (two items), negative DC (four items), (general) common DC (five items), including emotion- (two items) and problem-focused (three items) common DC. The psychometric properties of the DCI were considered good. Internal consistency (Cronbach’s alpha) of the DCI ranged between *α* = 0.71 and *α* = 0.93.

#### Hospital anxiety and depression scale

The Hospital Anxiety and Depression Scale (HADS, 32) is a 14-item self-report questionnaire measuring states of anxiety (HADS-A) and depression (HADS-D) on a 4-point response scale ranging from 0 (*not at all*) to 3 (*very often*). The HADS was originally developed from a study in the outpatient clinic of a general medical hospital. The license to use the HADS was obtained by the University of Zurich. In the current study, Cronbach’s alpha for the HADS was *α* = 0.87.

#### World Health Organization quality of life questionnaire

The *World Health Organization Quality of Life Questionnaire* (WHOQOL-BREF, 33 *)* is a widely used 26-item self-report instrument, not related to a specific disease and used to measure subjective quality of life. Based on a 5-point Likert Scale, the WHOQOL-BREF comprises the domains of physical and psychological health, social relationships, environment, as well as overall quality of life and general health. In the current study, internal consistency (Cronbach’s alpha) ranged from *α* = 0.58 to *α* = 0.81.

### Data analyses

The demographic data were analyzed and summarized with descriptive statistics in SPSS 25.0. Considering the normal distribution and dyadic structure of the data, paired sample *t*-tests were used to compare levels of anxiety, depression and quality of life between patients and their partners. Given the dyadic nature of the data, the Actor-Partner Interdependence Model (APIM, 34) was used to study the actor and partner effects of DC on anxiety, depression, and quality of life. An actor effect assesses, how an individual’s independent variable influences their own dependent variable (e.g., how DC of subjects with ESD relate to their own depression). A partner effect examines the influence of one dyad member’s independent variable on the dependent variable of the other member (e.g., how DC of subjects with ESD relate to the depressive symptoms of their caregiving partners). We calculated a *post-hoc* power analysis for the sample size of *N* = 37 couples for correlations assuming a medium effect. The test power (1−*β*) was 74%, which is just below the commonly accepted threshold of 80% for sufficient power. Covariances between causal variables and residual correlations between outcome variables of patients and partners for each DC domain were calculated and listed in **Table 2**. Data were analysed with LAVAAN in R, Version 4.3.3. A significance level of *p* <.05 was used.

**Table 2:** Covariances between causal variables and residual correlations between outcome variables of patients with ESD and their partners for each DC domain.

Outcome variable	DC domain							
		SC	SDC	DDC	NDC	CDC	CDCef	CDCpf
	Covariance between causal variables	0.16	-0.03	-0.23	0.00	0.039	0.45	0.37
Residual correlations between outcome variables of patients and partners for each DC domain								
Anxiety	0.05	0.05	0.05	0.04	0.05	0.06	0.04
Depression	0.00	0.02	0.02	0.01	0.02	0.02	0.01
QoL-physical	-0.04	0.00	-0.02	-0.04	-0.02	-0.02	-0.04
QoL-psychological	0.05	0.08	0.07	0.07	0.05	0.05	0.05
QoL-social	0.17	0.14	0.15	0.15	0.14	0.15	0.14
QoL-environmental	0.05	0.06	0.05	0.05	0.06	0.06	0.05
QoL-overall	-0.04	-0.03	-0.07	-0.04	-0.02	-0.02	-0.05

ESD, early-stage dementia; DC, dyadic coping; SC, stress communication; SDC, supportive DC; DDC, delegated DC; NDC, negative DC; CDC, common DC; CDCef, emotion-focused common DC; CDCpf, problem-focused common DC; QoL, quality of life.

To investigate potential relationships, Pearson correlations were calculated between education level, disease severity, anxiety, depression, and quality of life. The analysis revealed no significant correlations, suggesting that education level and disease severity did not substantially influence the associations observed between DC and mental health or quality of life outcomes. Consequently, these factors were not included as control variables in subsequent analyses.

## Results

### Comparison of DCI domains and anxiety, depression and quality of life domains

The comparison of anxiety, depression and quality of life domains between subjects with ESD and their partners revealed several significant differences. Patients reported significantly higher levels of stress communication compared to their partners (*t* (36) = 2.75, *p* = .009). Caregiving partners exhibited significantly higher levels of delegated DC than patients (*t* (36) = -3.44, *p* = .001). Patients showed significantly higher levels of emotion-focused common DC compared to their partners (*t* (36) = 2.64, *p* = .012).

An analysis of differences in anxiety, depression, and physical, psychological, social, environmental, and overall domains of quality of life showed that persons with ESD exhibited significantly lower anxiety (*t* (36) = -3.17, *p* = .003) and depression scores (*t* (36) = -2.07, *p* = .045) than their partners. No other statistical differences between both groups were found. The results are shown in **Table 3**.

**Table 3:** Differences in DC domains and outcome variables compared between patients with ESD and their partners (N = 37 couples).

Variables	Patients		Partners			
	*M*	*SD*	*M*	*SD*	*t*	*p*
SC	3.28	.95	2.79	.77	2.75	.009**
SDC	3.59	.83	3.83	.66	-1.36	.182
DDC	3.62	.99	4.39	.64	-3.44	.001**
NDC	2.22	.81	1.90	.77	1.73	.092
CDC	3.50	.82	3.17	.99	2.18	.036*
CDCef	3.23	.99	2.73	1.14	2.64	.012*
CDCpf	3.91	083	3.84	.92	0.47	.644
Anxiety	4.51	3.19	6.84	3.69	-3.17	.003**
Depression	3.68	2.69	5.11	3.20	-2.07	.045*
QoL-physical	60.71	9.85	59.94	7.68	0.34	.733
QoL-psychological	70.61	10.80	69.37	8.23	0.65	.521
QoL-social	74.77	18.69	71.40	17.95	1.15	.260
QoL-environmental	68.99	7.30	66.89	8.92	1.36	.182
QoL-overall	75.34	17.05	69.59	20.10	1.24	.223

DC, dyadic coping; *M*, mean; *SD*, standard deviation; SC, stress communication; SDC, supportive dyadic coping; DDC, delegated dyadic coping; NDC, negative dyadic coping; CDC, common dyadic coping; CDCef, emotion-focused CDC; CDCpf, problem-focused CDC; QoL, Quality of Life.

** *p* <.01; * *p* <.05.

### The correlations between DC, anxiety and depression

The APIM analyses revealed several significant actor effects and no partner effects of DC subscales on anxiety and depression. The significant results are summarized for patients with ESD and their partners in **Table 4**.

**Table 4:** Correlations between DC, anxiety, and depression in patients with ESD and their partners.

Patients or partners	Effect Type	DC domain	Outcome variable	*β*	*p*
Patients	Actor	DDC	Anxiety (own)	-0.37	.027
Patients	Actor	CDC	Anxiety (own)	-0.38	.035
Patients	Actor	CDCpf	Anxiety (own)	-0.36	.043
Partners	Actor	NDC	Anxiety (own)	0.40	.009
Partners	Actor	NDC	Depression (own)	-0.36	.019
Partners	Actor	CDCef	Depression (own)	-0.41	.015

DC, dyadic coping; DDC, delegated DC; NDC, negative DC; CDC, common DC; CDCef, emotion-focused common DC; CDCpf, problem-focused common DC; QoL, quality of life.

### The correlations between DC and quality of life domains

The APIM analyses revealed several significant actor and partner effects of DC on quality of life domains. The significant results are summarized for patients with ESD and their caregiving partners in **Table 5**.

**Table 5:** Significant actor and partner effects in and between patients and caregiving partners.

Patients or partners	Effect type	DC domain	Outcome variable	*β*	*p*
Patients	Actor	DDC	QoL-physical (own)	0.33	.049
Patients	Actor	CDCef	QoL-physical (own)	0.34	.047
Patients	Actor	SDC	QoL-psychological (own)	0.36	.018
Patients	Actor	CDC	QoL-psychological (own)	0.40	.023
Patients	Actor	CDCef	QoL-psychological (own)	0.39	.020
Patients	Actor	SDC	QoL-social (own)	0.35	.020
Patients	Actor	CDC	QoL-social (own)	0.38	.026
Patients	Actor	CDCpf	QoL-social (own)	0.39	.020
Patients	Actor	NDC	QoL-social (own)	-0.34	.026
Patients	Partner	SDC (Patients)	QoL-physical (Partners)	-0.39	.007
Patients	Partner	NDC (Patients)	QoL-overall (Partners)	-0.31	.026
Partners	Actor	NDC	QoL-psychological (own)	-0.33	.037
Partners	Actor	SC	QoL-social (own)	0.36	.020
Partners	Actor	SDC	QoL-social (own)	0.38	.012
Partners	Actor	DDC	QoL-social (own)	0.46	.003
Partners	Actor	CDCef	QoL-social (own)	0.35	.042
Partners	Partner	NDC	QoL-social (own)	-0.35	.022
Partners	Actor	SDC	QoL-environmental (own)	0.39	.012
Partners	Actor	NDC	QoL-environmental (own)	-0.42	.004
Partners	Actor	SDC	QoL-overall (own)	0.48	.001
Partners	Actor	CDC	QoL-overall (own)	0.40	.026
Partners	Actor	CDCef	QoL-overall (own)	0.39	.020
Partners	Partner	NDC	QoL-overall (Patients)	-0.45	.001
Partners	Partner	SDC	QoL-overall (Patients)	-0.36	.017
Partners	Partner	CDC	QoL-overall (Patients)	-0.36	.046
Partners	Partner	CDCef	QoL-overall (Patients)	-0.34	.045

DC, dyadic coping; SC, stress communication; SDC, supportive DC; DDC, delegated DC; NDC, negative DC; CDC, common DC; CDCef, emotion-focused common DC; CDCpf, problem-focused common DC; QoL, quality of life.

The overall variance explained by the APIM across all outcomes for both persons with ESD and their partners is 11%, indicating a moderate level of explanatory power. This result reveals a notable disparity between the model’s effectiveness for caregiving partners versus patients. While the model accounts for 15% of the variance in caregiving partners’ outcomes, it only explains 6% of the variance in the outcomes of persons with ESD. The most effectively explained variables are environmental quality of life for partners (21%) and overall quality of life for both groups (13% for patients, 30% for partners). Conversely, physical quality of life shows the weakest explanation for both patients (2%) and partners (2%). These findings suggest the potential influence of unmeasured factors on outcomes, particularly for persons with ESD.

## Discussion

This study examined the relationship between DC and anxiety, depression, and quality of life in couples facing ESD. While this study focused on the associations between DC and indicators of well-being, it is important to consider that the relationship is likely bidirectional. Not only can DC shape partners’ well-being, but individual levels of anxiety, depression, or quality of life may also influence how individuals engage in or perceive DC within the couple. The results showed the value of adopting a dyadic perspective when exploring coping with dementia, by recognizing that illness affects both partners and how each partner and the couple as a unit copes in their own particular way. A dyadic perspective uncovers the interpersonal dynamics that an individual approach tends to miss and is indispensable for understanding how couples respond to the demands of dementia in an early stage. The results reveal several new findings that contribute to our understanding of the role of DC in managing the challenges associated with ESD.

### Differences in DC, anxiety, depression and quality of life

Persons with ESD reported significantly higher levels of stress communication compared to their partners, which suggests that caregiving partners may be less likely to communicate their needs to their affected partners. This aligns with Bertschi et al. (35), who found that caregivers tend to refrain from communicating their stress to save patients from additional distress. This effect is known as ‘protective buffering’ (36). The effort to prevent patients from getting distressed over their declining performance provides one explanation why caregiving partners are inclined to take over many tasks and responsibilities early after diagnosis. A pragmatic consideration, which is avoiding the potential additional effort of cleaning up after, coordinating or supervising patients during task execution, may provide another explanation of increased delegated DC in partners (37). A compromised ability of subjects with ESD to contribute to practical tasks and shared problem-solving may explain their significantly higher levels of emotion-focused common DC in comparison with their partners. This suggests that they may engage more in joint emotional regulation strategies with their partners, whereas partners gravitate towards pragmatic solutions, whilst they downplay their own distress.

### Actor and partner effects related to anxiety and depression

The analysis revealed significant actor effects of DC on anxiety and depression.

As we expected, patients who provided more delegated DC and reported higher (general) common DC and problem-focused common DC showed lower anxiety, which may be due to a heightened sense of self-efficacy and participation. Being able to actively support a partner by taking over a task or effectively addressing a problem together may boost self-esteem and feelings of togetherness, maintain a sense of control and reduce feelings of helplessness and dependence (21, 37, 38).

When, for example, caregiver burden or feelings of uncertainty and helplessness give rise to more negative DC by caregiving partners, this may impact the relationship quality. Caregiving partners tend to experience higher levels of distress when they provide more negative DC. Negative reactions in patients, such as withdrawal or defensive responses, which altogether lowers the quality of the relationship, may feed into a process of mutual estrangement. On the contrary, when partners report engaging in joint emotion-regulation together, their individual distress is significantly lower. Whereas the well-being of subjects with ESD benefits from opportunities to participate and make contributions, their partners seem to benefit from maintaining a positive emotional connection to them.

However, the subjects in our sample were of high average age and the majority were retired. Factors leading to depression, anxiety and a reduced quality of life associated with seniority and retirement, include a loss of daily social contacts and diminished social participation, and a loss of professional identity, purpose and routine (39). Couples facing ESD in the current study may have been affected by these factors independently of the occurrence of a self-sufficiency reducing chronic illness in one of the partners. Thus, the distress resulting from an unsatisfied need for participation in persons with ESD and from a loss in emotional connection for partners may also be explained by the participants’ seniority and retirement status.

The absence of significant partner effects on anxiety and depression suggests that each partner’s DC behavior mainly impacted their own distress. However, the relatively small sample size could also account for the lack of partner effects found in relation to reports of anxiety and depression.

### Quality of life

With four partner and 19 actor effects, almost five times as many actor effects were found across various domains of quality of life. As we expected, psychological and social quality of life of patients with ESD was higher when they engaged more in supportive DC and common DC, including emotion-focused and problem-focused common DC, and less in negative DC. Providing support to their partner may increase the feeling of being valuable, boost their sense of autonomy and contribute to an experience of normalcy and social connection. Not surprisingly, patients who engaged more in criticizing or showing disinterest in their partner also reported lower social quality of life, which may be accounted for by poor relationship quality. A better physical quality of life in patients who take over tasks, may be well explained by higher levels of and more diverse physical activity, in comparison to those in a more passive and care-receiving role.

As for the patients, multiple relations were found between partners’ positive DC and their quality of life. Communicating distress, supporting the other in their problem-solving, helping out with tasks when needed may all contribute to a sense of connectedness and relationship quality. However, the caregiving partners were also found to communicate their distress less than patients, which may indicate that partners are holding back from sharing their experience of distress. The benefit of sharing distress is reported by Meier et al. (40), who discuss that partners who attempt to hide their own distress to avoid additionally upsetting their partners, may inadvertently cause greater distress within the couple. When both partners are impacted by a stressor, the use of common DC is more likely when the couple perceives the stressor as a shared challenge (41). Our results confirm these findings, demonstrating that emotion-focused joint coping effectively improves the quality of social life and lowers depression in caregiving partners.

Negative DC may reduce social engagement, negatively impacting both social and environmental living conditions for partners. Whereas feeling more in control may also encourage more constructive coping behavior, supportive DC may also contribute to a greater sense of control over the living environment, leading to improved environmental quality of life. Both general and emotion-focused common DC are likely to foster intimacy and relationship quality, and convey a sense of normalcy and continuity to the couple in the face of ESD related changes, contributing to an overall improved quality of life. Previous research confirmed an association between common DC and relationship quality in cancer couples (42). All found actor effects confirm our expectations – unlike the few partner effects found.

The partner effects entailed contra-intuitive results suggesting lower overall quality of life in patients, when their partners provided more positive (supportive, general and emotion-focused common) DC. Thus, whereas partners’ positive DC benefits their own quality of life, it appears to lower the quality of life of the patients. Possibly partners may tend more towards supportive DC, common DC and emotion-focused common DC, when patients show elevated levels of reflection and awareness, which might in turn increase the patients’ awareness of a restrained quality of life. The literature paints a mixed picture of the relation between DC and quality of life. On the one hand, our unexpected results conflict with findings in studies that found positive associations between DC and quality of life in couples facing physical chronic illnesses (e.g. 43, 44). On the other hand, research on couples facing dementia found that positive DC styles, such as supportive DC and common DC, are associated with more depressive symptoms in patients (17). In studies with healthy couples facing pregnancy, DC was positively associated with marital adjustment and quality of life (45, 46). Positive DC (47–50) and active engagement coping (51) were found associated with relationship quality, which is linked to quality of life (52). Furthermore, Hardy et al. (53) found ‘autonomy’ (i.e. a sense of volition in alignment with one’s values) to be associated with positive DC, and relationship satisfaction. Patients may experience supportive DC by their caregiving partners as a threat to their autonomy (17, 38), underlining the role of self-esteem in well-being in subjects with ESD. In spite of new insights presented in this study, the relation between DC styles and quality of life appears to reveal more nuance, beckoning for further research. **Table 6** presents a summary of the main findings and their explanation or interpretation.

**Table 6:** Summary table: dyadic coping, psychological outcomes, and quality of life in couples facing ESD.

Aspect	Finding	Explanation / interpretation
SC	Higher in persons with ESD than caregiving partners	Caregiving partners may suppress stress to protect the patient (protective buffering)
DDC(Partners)	Higher use associated with task takeover	Pragmatic or supportive motives; patients less able to contribute to shared tasks
CDCef (Patients)	Higher than partners	Patients may engage more in joint emotion regulation than problem-solving
Actor Effects on Anxiety (Patients)	Delegated DC, Common DC, Problem-focused Common DC → lower anxiety	Promotes self-efficacy, control, and active participation
Actor Effects on Anxiety/Depression (Partners)	Negative DC → higher anxiety; lower depression; lower CDCef	NDC reflects caregiver burden; CDCef reduces distress
Partner Effects on Anxiety/Depression	Mostly absent	Each partner's coping affects own distress; sample size may be limiting factor
Actor Effects on QoL (Patients)	Higher SDC, higher CDC, lower NDC	Participation boosts autonomy and social connectedness
Actor Effects on QoL (Partners)	Multiple positive links with SC, SDC and CDC	Reinforces emotional closeness, control, and intimacy
Partner Effects on QoL	Positive DC in partners → lower QoL in patients (contra-intuitive)	Possibly linked to increased patient awareness of loss, threat to autonomy
Age/Seniority	May confound anxiety, depression, QoL	Retirement-linked factors (loss of role, social contact) might explain distress

DC, dyadic coping; SC, stress communication; SDC, supportive DC; DDC, delegated DC; NDC, negative DC; CDC, common DC; CDCef, emotion-focused common DC; CDCpf, problem-focused common DC; QoL, quality of life.

Although dementia has a number of features in common with other chronic illnesses, it also differs in cognitive and interpersonal respects. Like cardiovascular diseases, COPD, cancer, and Parkinson’s disease, dementia also is a progressive syndrome that typically develops over many years, and ultimately leads to significant functional impairment (54). In all of these conditions, couples experience heightened stress and changes in relationship dynamics that necessitate adopting effective DC strategies (19, 22). For example, studies on couples facing cancer and COPD highlight that positive DC, including open communication and collaborative problem-solving, are associated with better relationship quality and psychological well-being for both partners (40, 42, 43). Similarly, the present findings show that supportive and common DC can buffer distress and enhance quality of life in couples facing ESD.

However, the direct impairment of memory, executive function, and awareness in people with dementia compromises their ability to participate in shared problem-solving and stress management, which is less common in chronic somatic conditions like heart disease or COPD. Whereas couples facing physical illnesses may largely retain the capacity for mutual support, couples facing dementia often develop asymmetric coping roles, with the caregiving partner increasingly assuming responsibility for both practical tasks and coming emotionally to terms with the changes and challenges at hand (17, 35). This can lead to partners withholding their own distress from people with dementia to not upset them, whereas this protective buffering is less common in, for instance, couples facing cancer (36, 42). Moreover, while positive DC often predicts improved quality of life in couples managing other chronic diseases, research in dementia sometimes reveals counterintuitive effects. For example, scholars reported increased depressive symptoms in people with dementia when partners provide more positive DC (17), which was also confirmed by our findings, possibly reflecting heightened disease awareness and autonomy loss. Thus, in spite of commonalities, the distinctive cognitive, emotional, and relational challenges of couples facing dementia differ from couples facing other chronic conditions, highlighting the need for tailored interventions and further comparative research.

### Limitations and future research

Several limitations of this study should be considered when interpreting the results. The study might be underpowered for detecting small-to-medium effects as a result of the relatively small sample size (*N* = 37 couples). As a result, the increased risk of Type II errors implies that significant differences and effects may not have been detected. Future studies with larger samples are needed to confirm and extend our findings.

The high age and retirement status made the study participants to experience heightened psychological distress, independently of the dementia affecting one of the partners. The role of socioeconomic status should be structurally assessed and controlled for in future studies. Ruling out the mere influence of being retired and of high age may help isolate the role ESD plays in the couple’s DC.

Given the cross-sectional nature of our data, we cannot draw conclusions about causal directions. It is equally plausible that lower well-being may reduce partners’ ability to engage in constructive DC, or that relationship quality moderates this dynamic interplay over time. The differential impact of DC on various quality of life domains suggests a multifaceted nature of well-being in the context of ESD. Longitudinal and cross-lagged studies could further elucidate the directionality of the relationship between DC and well-being and help identify patterns in the emotional and relational adjustments of couples facing ESD. In-depth qualitative studies could provide richer insights into the lived experiences of ESD couples and their DC. Examining DC patterns and outcomes over time as dementia progresses may add to our understanding of the process of couples’ dyadic adaptation to ESD-related challenges.

Furthermore, the study was limited to a sample of heterosexual couples in Switzerland, potentially limiting the generalizability of findings to other cultural contexts or relationship types. Future research should explore DC in diverse populations and relationship structures.

The reliance on self-report measures may introduce bias, particularly for subjects with ESD who may have an obstructed reflective insight into their own behaviors or experiences. Incorporating observational measures of DC could provide a more comprehensive understanding of couple interactions and support methodological validity.

Lastly, the moderate level of variance explained suggests that other factors not measured in this study may play important roles in determining anxiety, depression, and quality of life in ESD couples. The stronger explanatory power of our model for the caregiving partners’ outcomes (15.3% variance explained) compared to the patients’ outcomes (6.3%) suggests that factors beyond DC affect the well-being of patients with ESD in particular. These could include aspects such as communicative abilities, the sense of autonomy, the level of dependence, the availability of social support and the progression of cognitive decline. Future studies may wish to take these biopsychosocial aspects into account.

### Clinical implications

The findings of this study have several important clinical implications for supporting couples facing ESD. DC is correlated with self-efficacy, anxiety, depression, marital quality, and quality of life in patients with mild cognitive impairment (MCI) and their spouses (38). In the early stage of dementia, the effect of DC on anxiety and depression in our sample is limited, with no depressive symptoms found in patients. The predominance of effects of DC on quality of life when dementia is still in an early stage, may indicate that changes in domains of quality of life precede and predict clinical changes in mental health. Considering the progressive nature and prevalence of depression in couples facing dementia, preventative training programs should be implemented in an early stage. For instance, programs like The Couples Coping Enhancement Training (55) or the Daily Enhancement of Meaningful Activity Program (38) could help couples learn DC strategies adapted to ESD early on.

The different pattern of DC and well-being in couples facing ESD asks for interventions, tailored to address the specific needs of each partner. For caregiving partners, learning how to delegate care tasks, how to provide supportive DC without threatening patients’ sense of autonomy, how to avoid negative DC when dealing with distress or sharing their distress, and importantly, invest in an emotional connection with patients and the relationship quality helps improve quality of life and reduce mental health risks. Apart from accepting external care or emotional support from family, friends or professional services, clinical interventions may wish to teach couples a conjoint renegotiation of tasks and responsibilities. Couples that learn how to explore and discuss the redistribution of tasks together, what tasks or intermediate steps in tasks, affected subjects are still capable of and under what conditions, could build feelings of self-efficacy, reduce partners’ workload, and induce a sense of emotional connection, normalcy, and joint problem-solving in the face of individual and shared challenges.

## Conclusion

This study examined DC in couples facing early-stage dementia (ESD), focusing on its relationship with anxiety, depression, and quality of life. The research revealed significant actor effects of DC on these outcomes, particularly for quality of life domains. Patients with ESD providing more delegated DC and engaging in common DC showed less anxiety. Caregiving partners experienced less distress when engaging in emotion-focused common DC. ‘Positive’ DC from partners was associated with lower quality of life in patients. The study found more actor effects than partner effects, suggesting that DC behaviors primarily impact one’s own well-being. These findings highlight the lack of complementarity in coping among ESD couples and emphasize the need for tailored interventions that consider the unique needs of both partners.

## Data Availability

The raw data supporting the conclusions of this article will be made available by the authors, without undue reservation.
